# Intersecting motivations for leaving abusive relationships, substance abuse, and transactional sex among HIV high-risk women

**DOI:** 10.21633/jgpha.6.2s18

**Published:** 2016

**Authors:** Naomi S. David, Sophia A. Hussen, Dawn L. Comeau, Ameeta S. Kalokhe

**Affiliations:** 1 Rollins School of Public Health, Emory University, Atlanta, GA; 2 Rollins School of Public Health and Emory University School of Medicine, Emory University, Atlanta, GA; 3 Rollins School of Public Health, Emory University, Atlanta, GA; 4 Emory University School of Medicine and Rollins School of Public Health, Emory University, Atlanta, GA

**Keywords:** HIV prevention, intimate partner violence, drug use, transactional sex, HIV risk

## Abstract

**Background:**

Women bear a significant burden of the HIV epidemic in the United States. Women classified as ‘HIV high-risk’ often bring co-existing histories of intimate partner violence (IPV), drug use, and transactional sex. To help inform future comprehensive HIV prevention strategies, we aimed to explore common motivating reasons and barriers to leaving and/or terminating engagement in each of these risk-promoting situations.

**Methods:**

Between August and November 2014, in-depth interviews were conducted with 14 HIV high-risk women in Atlanta, Georgia who had experienced IPV in the previous 12 months, and used drugs and/or engaged in transactional sex in the previous five years. Participants were asked about histories of IPV, drug use, and/or engagement in transactional sex, and the motivating reasons and barriers to terminating each.

**Results:**

Women reported a range of motivating reasons for leaving IPV, drug use, and transactional sex. Overlapping themes included impact on children, personal physical health/safety, and life dissatisfaction. Financial need was identified as a common barrier to leaving.

**Conclusions:**

Future HIV prevention research should further explore the perceived impact of IPV, drug use, and transactional sex on physical health/safety, life dissatisfaction, one’s children, and financial need as motivators and barriers to reducing upstream HIV risk.

## INTRODUCTION

**Figure 1:**
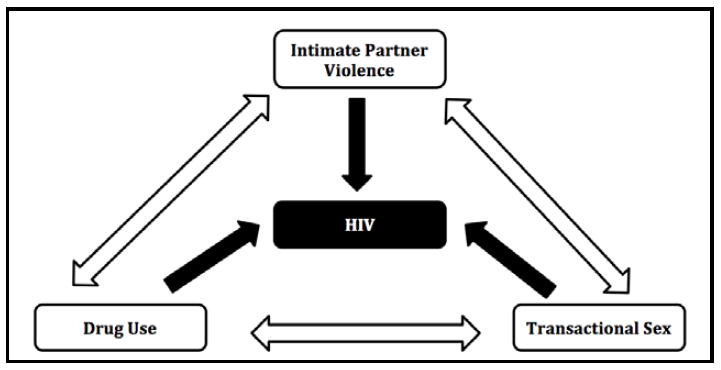
Complex interactions between IPV, drug use, transactional sex, and HIV

In the United States, women account for approximately 20% of new HIV infections, with the most common route of acquisition being heterosexual sexual intercourse (87%) (CDC 2015). Individually, the experience of intimate partner violence (IPV), substance abuse, and engagement in transactional sex contribute to the HIV epidemic among women, in part by increasing risk of sexual HIV acquisition (Dunkle and Decker 2013; Martin et al., 1999; Silverman et al., 2008; Baral et al., 2012; Kuo et al., 2011; Edlin et al., 1994; Chiasson et al., 1991; CDC, 2015). Additionally, these proximal risk factors often co-exist among HIV high-risk women and have a compounded effect on HIV susceptibility and concurrently serve as obstacles to accessing HIV prevention efforts (Figure 1) (Meyer et al., 2011; Singer et al., 2006; Singer 1994; El-Bassel et al., 2005; Batchelder et al., 2016).

Several pathways link IPV, drug use, and transactional sex to increased HIV risk. Women experiencing IPV are more likely to engage in condomless sex, use illicit drugs, have less control over safer sex practices, multiple and high-risk partners, and higher rates of sexually transmitted infections (STIs) (Molitor et al., 2000; Kilpatrick et al., 1997; CDC 2014; Silverman et al., 2011). Women who use cocaine or crack cocaine, the most common hard drug of abuse, are more likely to engage in risky sexual behaviors, in part due to impaired judgment and drug-induced impulsive sexual behavior (Lejuez et al., 2005). Women who engage in transactional sex are also more likely to have condomless sexual intercourse, multiple high-risk partners, and STIs, and neglect to undergo STI testing and treatment (Dunkle and Decker, 2013; Shannon et al., 2009; Wurth et al., 2013). Therefore, the high frequency of reporting and co-reporting of IPV experience, substance abuse, and transactional sex, along with the compounded effect on HIV risk, demonstrate the need for HIV prevention interventions for women to address the effects of these upstream risk factors.

This study, conducted in Atlanta, Georgia, an epicenter of the current U.S. AIDS epidemic, provides a unique opportunity to qualitatively explore common motivating reasons for and barriers to leaving abusive relationships and terminating engagement in substance abuse and transactional sex among HIV high-risk women. Knowledge of mutual motivating reasons and barriers could ultimately help inform the development of streamlined HIV prevention interventions for this difficult-to-reach population.

## METHODS

### Study Design

This qualitative study was the second phase of a study exploring the biologic link between IPV experience and HIV susceptibility (Kalokhe et al., 2016). During Phase I, participants completed a structured questionnaire about demographics, mental and physical health, IPV, and HIV risk behaviors. Upon preliminary review of Phase I data, substantial overlap in affirmative responses to questions about IPV, substance abuse, and transactional sex was apparent; thus, Phase II was designed to qualitatively explore these experiences. In Phase II, we aimed to investigate motivating reasons for and barriers to leaving IPV and terminating drug use and engagement in transactional sex. For this study, we defined ‘motivating reasons’ as self-identified rationalizing explanations for each of the questioned behaviors (Davidson 1963; Smith 1994). Interviews were conducted one-on-one in private clinic rooms of an infectious diseases center that serves over 5,600 HIV-infected individuals in Metro Atlanta.

### Eligibility and Participant Recruitment

Eighty-five HIV-negative, high-risk women aged 18-50 were recruited into Phase I of the study. “HIV high-risk” was defined using criteria from the Women’s Interagency HIV Study (Bacon et al., 2005; Kalokhe et al., 2016). All Phase I participants that met inclusion criteria were invited to participate; eligibility criteria for Phase II included report of past-year IPV experience and past 5-year transactional sex and/or hard drug use. IPV experience cut-points included a score of 50 or higher on the Index of Psychological Abuse (a measure of psychological IPV) and/or a score of 57 or higher on the Severity of Violence Against Women Scale (a measure of physical and sexual IPV) (Marshall 1992; Sullivan et al. 1992). These criteria aimed to capture subjects with more severe and/or frequent IPV exposure.

### In-depth Interviews

In-depth interviews were conducted and audio-recorded after obtaining written informed consent. The semi-structured interview guide explored past and present experiences with IPV, drug use, and transactional sex and motivating reasons, challenges, and barriers to leaving each. The interviewer (NSD) then worked with the participant to create a timeline of significant events, including experiences with IPV, drug use, transactional sex, and other traumatic life events (i.e. imprisonment and death of a parent) to provide additional context to their responses.

### Analysis

Coding and analysis were conducted utilizing a thematic analysis approach. Audio-recordings of the interviews were transcribed verbatim and the transcripts were imported into MAXQDA11© qualitative software for coding and analysis. The initial analysis occurred concurrently with the conduct of interviews. A codebook was generated from initial interviews. The codebook and definitions were reviewed and edited by members of the study team (NSD, ASK and SAH) and remaining transcripts were subsequently coded. Pseudonyms were assigned to participants to protect confidentiality.

### Participant Safety

The Emory University Institutional Review Board and Grady Health Systems Research Oversight Committee provided study approval. A regional domestic violence organization took part in the training of study staff about the sensitive conduct of IPV research. In line with World Health Organization guidelines, study flyers and staff referred to the study as a general health study for women during recruitment to conceal the true nature of the study from potential IPV perpetrators (WHO 2001). Subjects were notified of the true intent of the study during the consent process, and were provided a list of IPV community support services concealed in a phone book; study staff were available to facilitate referrals. All data and transcripts were de-identified and stored on a password-protected secure server.

## RESULTS

### Participant Characteristics

Between October and December 2014, 14 women participated in Phase 2 (Table 1). During the interviews, several reported experiencing a childhood traumatic event, such as abuse. Several attributed their initiation of drug use to the event and described drugs as means of coping. Many also reported periods of incarceration and homelessness, particularly while using drugs and selling sex.

### Motivating Reasons for Leaving a Violent Relationship

Participants were asked to identify motivating reasons for leaving prior violent relationship(s), and/or what it would take for them to leave their current abusive relationship. Some responses included a general overview of why they wanted to end their relationship, while others commented on the ‘tipping point,’ or final motivator that led to them wanting to leave (Table 2).

#### Escalation of violence

Eight of the 14 women identified an incident of escalated abuse that motivated them to leave their relationship. These events ranged from receiving a black eye due to physical assault, attempted murder with a firearm, and stabbing with knives or other objects. Jen, a currently homeless mother of five, decided to leave her partner after he attempted to murder her. She explains:

“Yeah one day he threw me in his car like ‘I’m gonna throw you in a ditch, bitch, and kill you’ and other shit and that was just a little bit too much. When I got away that last time I promised myself like…I didn’t want to be beat.” (Jen, age 35)

Women also described their partner’s increased controlling behaviors as a trigger to leave the relationship. For example, some partners reprimanded the women for leaving the house, while others displayed intense rage and jealousy, including false accusations of infidelity. Christine describes her controlling boyfriend:

“And it got worse, it didn’t get no better. He started to the point where he wanted to lock me in the house, beat me up…I thought I was an old—I could’ve sworn I was about 50 years old, how he made me feel.” (Christine, age 33)

#### Tired of abuse and desire for a better life

There was overlap in women discussing feeling tired of abuse and wanting a better life. Some spoke generally about wanting more for themselves and their families, and others came to realize they would be better off without their violent partner. Caroline spoke about the emotional abuse and her realization that the condition was not going to change. She explained, “I just got to the point where I wanted more for myself. I finally started to care about myself…I just finally said ‘you know what, I need to do something for me’.” (Caroline, age 41)

Renee concluded that the abuse was not going to stop and that only she could remove herself from the relationship. Ashley wanted to be free of the abuse and drug-using lifestyle, and Marissa grew tired of her alcoholic husband and desired an equal financial contributor, as well as a violence-free home. Additionally, several women described hopelessness and apathy at the height of their abuse before deciding to leave.

#### Children

Two women said their children were the impetus for leaving an abusive relationship. Anna described the shame she experienced when her partner displayed aggression in front of her children. Likewise, Ashley commented on the importance of setting a positive example for her daughter rather than remaining in a violent household, “I was not going to let my daughter grow up to think that it’s alright for someone to call her a name or be disrespectful or put their hand on them.” (Ashley, age 42)

### Barriers to Leaving Violent Relationships

Major themes included needing shelter, lacking the financial means to sustain themselves, or seeing a move as interfering with their children’s wellbeing. Anna was contemplating leaving her partner and commented:

“It’s a matter of finding a place [to live] for me and my four kids, and I’m currently seeking employment…it was my mistake by allowing him to [financially] handicap me and me becoming co-dependent upon him because I wasn’t working. I’m going to seek employment and try to save a little money and then move.” (Anna, age 30)

Additionally, some women reported fear of retaliation from their partners as a major barrier to leaving. Some reported partners restraining them from leaving the house, or resorting to hiding from their partners and sneaking belongings out of shared living spaces. Leaving homes safely with their possessions was a major challenge for some. For example, Marissa had to ultimately make a choice between her safety and possessions:

“The only struggle was holding on to my belongings, but eventually I just said screw it, that’s just material.” (Marissa, age 38)

### Motivating Reasons for Terminating Drug Use

Twelve participants reported use of an illicit hard substance (i.e., crack, cocaine, methamphetamines, heroin) in the previous five years, and 6 are currently using crack/cocaine. Women reported a range from infrequent “social” use among friends, to everyday use and addiction for several years. Their motivations for terminating drug use or factors that would motivate them to stop in the future are shown in Table 2.

#### Children

Eight of the 12 women cited a motivating reason for terminating drug use that involved their children. Women described the need to set a better example for their children, longing to be a ‘better parent,’ and desire to provide the best for their children. Renee mentioned the need for her four-year-old daughter’s approval who was unhappy with her mother smoking ‘cigarettes’ [marijuana]. Jessica stated her daughter did not want to be in her presence because of the effects of Jessica’s drug use on her irritability. Sylvie reported how the grief of losing her child prompted her to further focus on her children and motivated her to quit using drugs:

“Well you know when my son died with cancer at 4 years old—it kinda’ turned my life around and it took a toll on me, you know. Where I didn’t want to do any more crack, you know. Everything, you know it just took a big toll on me ‘cause my kids are my everything and it just shut me down.” (Sylvie, age 45)

Susanne, Christine, and Caroline lost legal custody of their children and wanted to stop using drugs so that their children could live with them again. Caroline describes her inability to properly care for her children when she was using. She said:

“I couldn’t function [on drugs], I couldn’t be stable…I couldn’t be a good mom. I tried, and I love my kids, I didn’t abuse them by neglecting, I didn’t whip them or anything like that but um just by not being there.” (Caroline, age 41)

Another participant, Ashley, had delivered two babies who tested positive for cocaine when they were born. During her third pregnancy, she was determined to have a child that tested negative for cocaine. She recalls:

“I went back to rehab when I found out I was pregnant again with my daughter. And um, so I took two of my kids through [pregnancy] getting high…I had two positive children come out with cocaine in their system and I promised myself I wasn’t going to let her come out with that.” (Ashley, age 42)

#### Adverse health effects

Five women described fear of negative health consequences as a deterrent to drug use. In particular, they discussed addiction, overdose, death, and physical pain. Christine referenced her fear of dying from an overdose. She explains, “[I’m afraid of] harming my body, making me think that if I do too much it’ll kill me” (Christine, age 33). Jessica describes the negative impact of withdrawal on her mental health when she attempted to come off of drugs and implied that it interfered with her concentration and worsened her temperament.

#### Sober life and self-betterment

An additional theme was the desire for women to better themselves as a motivation to stop using drugs. Some women mentioned that they always figured they would stop drugs at some point, and that they would like to have a sober lifestyle. Lynn reflected, “I just got tired of that life [homeless and using drugs]…the hustle and bustle, hustling, getting out of jail, just not having your own place to live” (Lynn, age 49). Nicole, a former addict, describes her decision to stay clean after being in prison. In particular, she described the new and previously unknown benefits of sobriety and the positive outcomes on her family life. She explained:

“It was a decision. I was either going to continue to get high or not get high…I knew what it was like with the tricking off and the prostitution and the sucking penises, the stealing, the manipulation… I never knew what it was like to be clean, be sober, wake in the morning …so I wanted to try, and now I like it.” (Nicole, age 33)

Some women mentioned the desire for the stability of a sober lifestyle, that abstinence could improve personal relationships, financial status, their fulfillment of family and work responsibilities, and life satisfaction.

#### Financial cost

Three women mentioned wanting to terminate their drug use to avoid the financial cost of drugs, stating that it was challenging to pay for routine household expenses and maintain their drug use. While referring to cocaine, Christine comments:

“Don’t start doing this [using cocaine] if you don’t have the money for it. And sometimes you gonna spend your last [dollar] to go get it…I had to start working on that because now I have bills, and I refuse to be homeless.” (Christine, age 33)

### Barriers to Terminating Drug Use

Some participants identified associating with their social groups or intimate partners as a barrier to quitting drugs. They found it challenging to abstain when their friends and partner(s) were using drugs in their presence, and severing such social ties was not desirable. Several women spoke about the overwhelming physical pain of withdrawal and that their previous experiences of withdrawal diminished their likelihood of quitting drugs. Christine described, “your body don’t feel the same, because it’s [drugs] in your blood. It’s in your system. So it’s like your system constantly doing this 360….and when you stop trying to do stuff [drugs and/or alcohol], I get sick.” (Christine, age 33)

### Motivating Reasons for Terminating Engagement in Transactional Sex

Thirteen of the 14 participants had a history of providing sex in exchange for money, drugs, shelter, or other necessities. There was variety in the number of transactional sex partners reported, the type of transactional relationship (i.e. stranger, friend, intimate partner, etc.), and the sexual activities described in these relationships (oral sex, vaginal sex, anal penetration with object, etc.). Additionally, participants viewed their engagement in transactional sex differently; some did not consider sex in exchange for money as ‘selling sex,’ particularly if they were in a romantic relationship. The participants’ motivating reasons for terminating engagement in transactional sex are listed in Table 2.

#### Sexual health

Several participants expressed concerns about acquiring STIs, with five directly mentioning STI risk as a motivating factor to stop selling sex. Jen describes her safety concerns in the following quote, “There’s so much disease…you just touch somebody and catch something—it’s just not safe [transactional sex]. You know people’s mouths and stuff…I just don’t want that type of company no more.” (Jen, age 35)

#### Physical safety

In addition to concerns for sexual health risks, women also reported apprehension of the physical safety risks associated with selling sex. Four participants identified concerns for safety (i.e., assault, rape, death) as a motivation for terminating engagement in transactional sex. Jessica describes her fear:

“It’s too risky because of course they [clients] can’t come to my home, so I’d have to meet them somewhere—their home, or a hotel…. I’ve seen a lot, you know I watch the news—these girls are you know, getting set up, or rocked [assaulted] and shot, or getting raped.” (Jessica, age 36)

#### Desire for monogamy

Four women reported a desire to terminate transactional sex to maintain a monogamous relationship with their partner. Kim stated she would stop selling sex if she and her girlfriend were to marry. Marissa described how she stopped selling sex when she married to remain faithful to her husband. Jessica, who started selling sex for income upon her release from prison, described becoming more serious with her current boyfriend as her decision for stopping. Anna, who has sold sex throughout her current relationship, desires to stop selling sex to avoid having excess partners, and because she desires a loving relationship with her sexual partner.

#### Dissatisfaction with selling

Four women reported being dissatisfied with selling and sought self-betterment or life satisfaction. Some of the women spoke of transactional sex as a ‘lifestyle,’ particularly when they were using drugs and selling sex simultaneously. For some, this lifestyle was a negative situation that they wished to escape, while others viewed it as a period of bad decisions that was contingent on a drug addiction. Caroline describes her dissatisfaction with selling sex in the following quote:

“It’s been years [since selling sex] because I’m not at that state that I used to be…I kind of respect myself today. And I think like being out there like that [selling sex] and now I’m very particular, like I can’t just be with anybody…I kind of just respect myself more.” (Caroline, age 41)

### Barriers for Terminating Engagement in Transactional Sex

Financial need was a commonly cited barrier to terminating engagement in transactional sex. The women generally spoke of transactional sex as a choice they made to survive, or as a last resort. Sylvie described how household and family expenses fueled her since she doesn’t have an income. Similarly, Anna describes that selling sex is not the ideal situation; when asked if she foresees selling sex in the future, she responds with the following:

“This is not an occupation. It’s not a hobby, its not something I just do all the time, but I always know if push comes to shove it’s something I could convert to or something…It’s not a good one [option] but nevertheless, it’s there.” (Anna, age 30)

Like Anna, several of the women described the need for financial resources as a reason to continue with transactional sex even when they preferred not to engage in this behavior.

## DISCUSSION

To help streamline HIV prevention interventions for this difficult-to-reach, high-risk population, this study sought to explore common motivations for and barriers to leaving abusive relationships and ceasing to engage in drug use and transactional sex. Across IPV, drug use, and transactional sex, a range of motivating reasons was identified for leaving or terminating each, with some overlap in responses. Children, life dissatisfaction, and concern for physical health and safety emerged as common themes, and financial need was identified as a mutual barrier.

### Children

Children were mentioned as a motivating reason for leaving IPV, drug use, and transactional sex. Previous research on mothers contemplating leaving an abusive relationship has identified children as both a motivating reason to leave and to stay (Rhodes et al. 2010; Chang et al. 2010; Campbell et al. 1998). The studies suggest that mothers factor the children’s safety, the effects of the child’s exposure to abuse, the comfort of their children, and desire to remain an ‘intact’ family in their decision. Our findings also align with the literature examining motivations for leaving transactional sex and drug use. In a mixed-methods study of female sex workers and their intimate partners, some couples identified their children’s wellbeing as a motivator for decreasing drug use and engagement in sex work, while others identified children and child-rearing costs as a motivator for continuing sex work (Rolon et al. 2013). Similarly, a study of female sex workers in Thailand found that the woman’s ability to cease sex work was primarily dependent on her finances, but that desire to hide her sex work from her children was also a factor (Manopaiboon et al. 2003). Our findings taken together with the literature suggest the perceived harm to a child’s physical or mental wellbeing and/or environment is a critical motivator for women to consider leaving abuse, drug use, and transactional sex.

### Physical Health and Safety

Negative effect on health and safety was a common motivating reason to leave abusive relationships and disengage in transactional sex and substance abuse. This finding is in accord with prior work. Other studies have also demonstrated that escalation of violence, such as introduction of a weapon, can be both a motivator to leave, and motivator to stay due to an escalation of fear (Lacey, Saunders, and Zhang, 2011; Campbell et al. 1998; Chang et al. 2010). In qualitative interviews with methamphetamine users, negative impact on physical health was a leading motivator to quit (German et al. 2006). Drug-related hospitalizations have also been described as motivating reasons for stopping cocaine, alcohol, and heroine (Nyamathi et al. 2004). Among sex workers, sexual health risks including HIV infection have previously been demonstrated to be a motivation for terminating transactional sex (Manopaiboon et al. 2003).

### Life Dissatisfaction

Dissatisfaction with their current lifestyle was also a common motivating reason across all three categories. Prior studies evaluating motivators reasons for leaving abusive relationships, substance abuse, and transactional sex independently have noted similar findings. For example, studies evaluating triggers for leaving abuse have identified ‘inability to endure the violence anymore’ as a key factor in leaving; for transactional sex, ‘deep hatred for the profession,’ ‘being tired of sex work,’ and ‘feeling disgraced by the nature of the work itself’ have been described (Manopaiboon et al. 2003; Haj-Yahia and Eldar-Avidan, 2001); and for substance abuse, not wanting ‘lives of addiction,’ perceiving self as ‘being dirt,’ and ‘wanting a better future’ (Meade et al. 2015; Sobell et al. 2001).

### Barriers for Leaving IPV, Drug Use, and Transactional Sex

**Figure 2:**
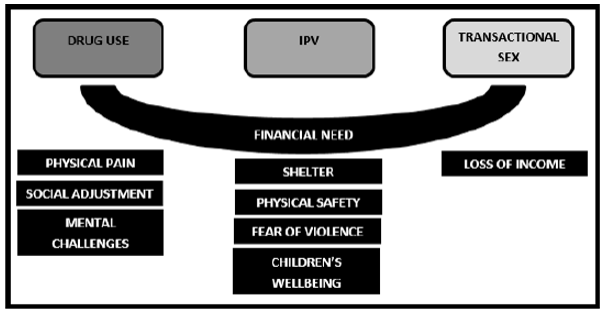
Barriers for leaving a violent relationship, terminating drug use, and transactional sex

There was little overlap in reported barriers for leaving a violent relationship, and terminating drug use and transactional sex (Figure 2). However, financial need was a mutually identified barrier to leaving IPV and transactional sex. Financial need meant women had to rely financially on their abusive partners and could not independently secure the necessary resources to find alternative shelter or provide for their children. Financial need was a barrier to terminating engagement in transactional sex because it resulted in a loss of income. Financial need was not explicitly identified as a barrier to terminating drug use, but poverty and homelessness were both discussed as motivating reasons to initiating and continuing drug use. Additionally, of the participants who successfully completed drug rehabilitation programs, all attended the programs free-of-charge and reported that they would not have overcome their addiction had it not been for the rehabilitation. It is possible that other women using drugs may desire to enroll in a drug rehabilitation program, but are limited financially; these conclusions are consistent with previous literature (CDC 2013; Anderson and Saunders, 2003; Manopaiboon et al. 2003).

### Implications for HIV Prevention Research

First, the overlapping motivations and barriers to leaving IPV, drug use, and transactional sex identified through this qualitative study need to be validated using a larger-scale quantitative design. Second, our study informs strategies to help streamline HIV prevention efforts for this HIV high-risk population. For example, an impact on children and personal health and safety were identified as motivating reasons for leaving all three HIV risk factors. Therefore, HIV prevention interventions for this population could focus on educating women about the negative social, physical, and mental health impact of staying in an abusive relationship, using drugs, and engaging in transactional sex on their children and selves. Additionally, financial need was identified as a common barrier to leaving IPV, drug use, and transactional sex; future HIV prevention interventions for this population might consider a component of financial empowerment. Similarly, interventions with a decision balance/cognitive evaluation approach could help participants evaluate the benefits of a ‘better life’ without abuse, drug use, or transactional sex (Sobell et al. 2001).

A limitation of this study is the potential recall bias, as participants were asked to reflect on both recent and remote events; recall bias may have resulted in women misremembering events or providing motivating reasons reflective of more recent or severe circumstances. Additionally, there is the potential for social desirability bias affecting participant responses; to reduce this, the authors aimed to create a safe space for the interview and emphasized the confidentiality and anonymity of participation. The single coder for the analysis may have also biased the results. We attempted to mitigate this effect through having three investigators review the codebook and having the coder discuss uncertainties with other co-investigators throughout the analysis.

## CONCLUSIONS

HIV high-risk women who experience IPV, drug use, and transactional sex are a critical, yet difficult, population for HIV prevention efforts to reach. By identifying common motivations and barriers for leaving each, our study is the first to shed light on elements critical for developing streamlined HIV prevention strategies for this key population. Future research should first validate the findings from this paper on a larger, quantitative scale, and then use them to develop HIV prevention interventions for women who concurrently experience IPV and engage in substance abuse and transactional sex.

## Figures and Tables

**Table 1:** Demographics Characteristics of Participants

Age Range	n (%)	Annual Household Income	n (%)
30-35	7 (50)	Less than $10,000	10 (71)
36-40	2 (14)	$10,000-$20,000	4 (29)
41-45	3 (21)	**Currently Employed**	
46-50	2 (14)	Yes	4 (29)
**Race**		No	10 (71)
African American or Black	14 (100)	**Sexual Orientation**	
**Education (highest level completed)**		Heterosexual	7 (50)
Some high school	4 (29)	Lesbian, gay, or bisexual	7 (50)
High school/GED graduate	5 (36)	**Childhood Abuse (age 17 or younger)**	
Beyond high school	5 (36)	Physical abuse	9 (64)
**Biological Child Count**		Sexual abuse	8 (57)
1-2	7 (50)	Emotional abuse	12 (86)
3-4	3 (21)	**Recent Substance Abuse**	
5-6	3 (21)	Cocaine/crack use (1+ in past week)	6 (43)
7+	1 (7)	Marijuana use (1+ in past week)	9 (64)
		Alcohol consumption (8+ in past week)	6 (43)

**Table 2:** Motivating reasons for leaving a violent relationship with an intimate partner, and terminating drug use, and engagement in transactional sex

Motivating reasons for leaving a violent relationship	No. (%) (n=14)	Motivating reasons for terminating drug use	No. (%) (n=12)	Motivating reasons for terminating engagement in transactional sex	No. (%) (n=12)

Escalation of abuse	8 (57)	Children	8 (67)	Sexual health (STI fear)	5 (42)
Desire for a better life	5 (36)	Adverse physical effects	5 (42)	Desire for monogamy	4 (33)
Tired of abuse	3 (21)	Self-betterment/sober life	4 (33)	Physical safety	4 (33)
Children	2 (14)	Financial cost	3 (25)	Dissatisfaction with selling	4 (33)
Family intervention	1 (7)	Rehabilitation	3 (25)	Monetary motivation resolved	2 (17)
		Neglected responsibilities	2 (17)	Children	1 (8)
		Intimate partner	2 (17)		
		Work performance	1 (8)		
		Family relationships	1 (8)		

* participants could report more than one response; total exceeds 100%
